# MiniBioReactor Array (MBRA) *in vitro* gut model: a reliable system to study microbiota-dependent response to antibiotic treatment

**DOI:** 10.1093/jacamr/dlac077

**Published:** 2022-07-05

**Authors:** C A Hobson, L Vigue, S Naimi, B Chassaing, M Magnan, S Bonacorsi, B Gachet, I El Meouche, A Birgy, O Tenaillon

**Affiliations:** IAME, UMR 1137, INSERM, Université de Paris, AP-HP, Paris, France; IAME, UMR 1137, INSERM, Université de Paris, AP-HP, Paris, France; INSERM U1016, Team ‘Mucosal Microbiota in Chronic Inflammatory diseases’, CNRS UMR 8104, Université de Paris, Paris, France; INSERM U1016, Team ‘Mucosal Microbiota in Chronic Inflammatory diseases’, CNRS UMR 8104, Université de Paris, Paris, France; IAME, UMR 1137, INSERM, Université de Paris, AP-HP, Paris, France; IAME, UMR 1137, INSERM, Université de Paris, AP-HP, Paris, France; Laboratoire de Microbiologie, Hôpital Robert Debré, AP-HP, 75019 Paris, France; IAME, UMR 1137, INSERM, Université de Paris, AP-HP, Paris, France; IAME, UMR 1137, INSERM, Université de Paris, AP-HP, Paris, France; IAME, UMR 1137, INSERM, Université de Paris, AP-HP, Paris, France; Laboratoire de Microbiologie, Hôpital Robert Debré, AP-HP, 75019 Paris, France; IAME, UMR 1137, INSERM, Université de Paris, AP-HP, Paris, France

## Abstract

**Background:**

Antimicrobial drugs are mostly studied for their impact on emergence of bacterial antibiotic resistance, but their impact on the gut microbiota is also of tremendous interest. *In vitro* gut models are important tools to study such complex drug–microbiota interactions in humans.

**Methods:**

The MiniBioReactor Array (MBRA) *in vitro* microbiota system; a single-stage continuous flow culture model, hosted in an anaerobic chamber; was used to evaluate the impact of three concentrations of a third-generation cephalosporin (ceftriaxone) on faecal microbiota from two healthy donors (treatment versus control: three replicates per condition). We conducted 16S microbiome profiling and analysed microbial richness, diversity and taxonomic changes. β-Lactamase activities were evaluated and correlated with the effects observed in the MBRA *in vitro* system.

**Results:**

The MBRA preserved each donor’s specificities, and differences between the donors were maintained through time. Before treatment, all faecal cultures belonging to the same donor were comparable in composition, richness, and diversity. Treatment with ceftriaxone was associated with a decrease in α-diversity, and an increase in β-diversity index, in a concentration-dependent manner. The maximum effect on diversity was observed after 72 h of treatment. Importantly, one donor had a stronger microbiota β-lactamase activity that was associated with a reduced impact of ceftriaxone on microbiota composition.

**Conclusions:**

MBRA can reliably mimic the intestinal microbiota and its modifications under antibiotic selective pressure. The impact of the treatment was donor- and concentration-dependent. We hypothesize these results could be explained, at least in part, by the differences in β-lactamase activity of the microbiota itself. Our results support the relevance and promise of the MBRA system to study drug–microbiota interactions.

## Introduction

Antibiotic resistance is preoccupying as it is associated with treatment failure in infected patients, and increased morbidity.^1,2^ The link between antibiotic consumption and antimicrobial resistance is largely admitted.^3^ Indeed, the effects of antibiotics goes beyond the infected site, they can destabilize the microorganisms hosted in the gut^1,4–6^ and increase the expression of resistance genes in gut bacteria through environmental pressure.^7^

The gut microbiota is an organ in itself, specific to each individual,^8–10^ implicated in metabolic activities, immune response regulations, barrier functions and colonization resistance, directly affecting the host’s health.^1,6,11–14^ Gut bacteria degrade and produce carbohydrates, short chain fatty acids (SCFAs), vitamins and amino acids,^15^ all with specific roles to maintain homeostasis in the host.^11,16–18^

Over the last decade, concern has been raised on the link between the impact of antibiotic consumption on the gut bacteria and human diseases.^19,20^ Disturbances in the gut microbiota composition (i.e. dysbiosis)^21^ are suspected to have a causative or aggravating role in many human diseases.^6,14,20^ The role of the gut microbiota is also important in therapeutics, as the host response to different therapies is influenced by the gut microbiota composition.^9,11^

Antibiotic exposure is responsible for decreased diversity in the faecal bacterial communities and modifications in the global composition resulting in dysbiosis.^5,6,14,20,22–24^ Antibiotic treatment can be associated with the selection of resistant clones and modification of the expression of resistance genes,^2,20,25^ a shift in the dominant clone,^7,26^ modifications in colonization resistance,^23,27^ stress-induced phenomena such as horizontal gene transfer, and increased mutagenesis,^28^ all responsible for increased antibiotic resistance.^7,29,30^

However, it is worth noting that the impact of antibiotic treatment on the gut microbiota interactions is variable depending on the host and highly related to the molecule, its dose, route of administration and pharmacokinetics/dynamics.^2,5,24^ One interesting hypothesis is that the gut microbiota itself could modulate the impact of antibiotic treatment and contribute largely to the inter-individual variability observed between treated individuals. Indeed, some microbes are more resistant than others to the antibiotics used, and some resistant microbes may even degrade the antibiotic to some extent, modifying its effective concentration in the gut. However, testing the potential role of microbiota composition on the impact of a treatment is complicated as hosts have not only different microbiota but also different physiology that could also contribute to the variable impact.^20^ Host factors such as the gut microbiota are highly suspected to mediate this interaction between antibiotic and bacteria. Indeed, the observed variability in the dynamics of spread of the same antibiotic-resistant strain between individuals suggests that the tryptic ‘antibiotic–bacteria–host’ interactions are implicated in the resistance dynamics.^31^

Human clinical trials suffer from limited sampling and replication due to cost, ethical reasons and gut composition variability through time.^5,24,32,33^ One individual cannot be in the treated group and the control group at the same time. Moreover, in all these *in vivo* models, it is hard to disentangle the contribution of the host to that of its microbiota.

Recently, several *in vitro* models have been proposed to use and expand a system that allows the maintenance of a complex ecosystem similar to the human gut.^34,35^ These models offer the advantage of numerous simultaneous replicates, in a controlled environment with controlled duration of experiments and unlimited screening conditions, enabling high-throughput experiments and limited ethical constraints.^34–38^  *In vitro* models lacking the complexity of the host physiology can be used to test the direct interactions between microbiota and treatment without any other confounding factors.^39–41^

One patient can be its own control throughout the experiment, and many conditions can be evaluated simultaneously on the same faecal sample allowing experimentation on the relationship between human pathology and therapeutics.^42–44^ Indeed, *in vitro* human gut models allow study of the specific relationship between drugs and the faecal microbiota, as, in these systems, all the other interfering parameters are ignored.^38,45–50^

The MiniBioReactor Array (MBRA) is a low-cost, high-throughput and reproducible system that has been developed recently.^51,52^ This system has been validated for culture of human faecal bacterial communities up to 3 weeks,^51^ and is used as a *Clostridioides* infection model.^52,53^ More recently, the impact of dietary emulsifiers on gut inflammation and perturbation using this system has been evaluated.^47^

We chose therefore to evaluate the impact of ceftriaxone, a third-generation cephalosporin with biliary excretion, on human gut microbiota combining the MBRA system with the 16S rRNA profiling method. Our main objective was to evaluate the reliability of the MBRA in mimicking the intestinal microbiota and its modifications under antibiotic selective pressure.

## Materials and methods

### Study design

We used the MBRA system, placed in an anaerobic chamber (CoyLab^®^) at 37°C to perform a study of the impact of ceftriaxone on gut microbiota.

### Faecal sampling and storage

Human faecal samples from two healthy adult donors (i.e. non-symptomatic state) belonging to the same age range were used. The two donors have no medical history, no history of any treatment for at least a year before the experiment. One is a female and the other a male, they do not live in the same environment. They have similar diets; they are not vegetarian nor vegan; and their fibre or fat intakes are comparable. Both donors are in the normal weight range for their age and height.

### Ethics

In agreement with the INSERM ethics regulation, and with the declaration of Helsinki, all received clear information and consented. At emission, the faeces were directly collected in a sterile anaerobic container, and subsequently stored at −80°C.

### Consent for publication

Not applicable.

### Impact of ceftriaxone on gut microbiota

Three concentrations of ceftriaxone (1 mg/L, C1; 10 mg/L, C10; and 100 mg/L, C100) and a negative control (no treatment, control) were evaluated in triplicate. The dose of treatment was injected once daily, and consecutively for 5 days.

### Experimental design

The MBRA consists of 24 independent chambers, containing a 15 mL culture volume, maintained by two peristaltic pumps, adapted for low flow rates.

We used the BRM medium that mimics composition of colonic gut as previously described.^47,51^

We followed Auchtung *et al.*’s protocol for sample preparation and faecal inoculation.^51^ The same faecal sample was used to inoculate 12 chambers for each donor. After a resting time of 16 h, the flow rate was 1.9 mL/h (equivalent to an 8 h turnover, mimicking the physiological intestinal peristalsis). Daily samplings were performed in the 24 chambers. The evolution of the 24 microcosms (treatment versus control × 1 drugs × 2 donors × 3 replicates per condition) was followed through the characterization of the 16S rRNA diversity. In total, we collected samples at different timepoints, and five key timepoints are represented: the equilibrium state obtained in the chambers on Day 3 (T3), 24 h after the first dose of treatment (T5), after three doses of treatment (T7), 24 h after the last dose (T9) and 72 h after the last dose of treatment (T11).^47^

### Sample processing and 16S rRNA gene sequencing

#### DNA extraction

We used the QIAamp 96 PowerFecal QIAcube HT Kit^®^ (Qiagen) following the manufacturer’s instructions, and subsequently performed DNA extraction with the QIAcube^®^ High-Throughput 96 samples robot.

#### 16S rRNA gene sequencing

16S rRNA gene sequencing was used to analyse the bacterial communities in our samples, targeting specifically the V4 hypervariable region.^54^ Libraries were prepared as specified by the manufacturer. Briefly, PCR 1 enables the amplification of the 16S region, using 515 forward primer and 806 reverse primer.^55^ PCR 2 corresponded to the multiplexing step of the samples on the 16S amplified regions, using the Nextera^®^ Index Kit and Nextera^®^ XT Index kit V2 Set D. The DNA was quantified with Qubit HS^®^ and normalized to 4 nM for pooling and sequencing.

#### Sequencing

We used the Illumina MiSeq technology (paired end reads 2 × 250 bp) for 16S rRNA gene sequencing.

### Data analysis

The microbiome bioinformatics analysis were performed with the QIIME2-2020.8 software.^56^ We denoised samples with the DADA2 (via q2-dada2) pipeline.^57^ We subsequently aligned reads with Mafft^58^(via q2-alignement) and constructed phylogenetic with fasttree-2 (via q2-phylogeny).^59^ α-Diversity (diversity within each chamber) was analysed with the Shannon and evenness indexes, β-diversity (diversity between each chamber) was represented by Jaccard and weighted UniFrac analysis (using q2-diversity). For diversity analysis, samples were prior rarefied to 7900 sequences per sample, therefore excluding two samples from our dataset. We assigned taxonomy to all amplicon sequence variants with a 99% threshold of pairwise identity, using a naive Bayes classifier trained on the Greengenes reference database 13_8 (using q2-feature-classifier).^60^ Counts are obtained from rarefied data. Last, we calculated the Firmicutes to Bacteroidetes (F/B) ratio, including all time during and after treatment. All generated data visualizations were performed using Python3. We used R version 1.2.5033 to analyse the data. The ‘change from baseline’ analyses were conducted considering T3, the stabilization time, as the baseline point.

### Measure of β-lactamase activity

β-Lactamase activity was evaluated on samples at T3 corresponding to the time just before the treatment (8 samples per donor in two replicates, *n* = 16 samples per donor). Briefly, a crude cell extract was obtained using β-LACTA^™^ (Bio-Rad) lysis reagent on T3-cultured microbiota. Lysates were diluted (1/10 times) in potassium phosphate buffer pH 7 containing BSA and nitrocefin (50 μg/mL), to evaluate penicillinase activity. Initial velocity was measured spectrophotometrically at 486 nm using a Tecan infinite 96-well plate reader at 37°C. From the OD measurement every 30 seconds, we calculated the relationship between the slope of the response curve and the enzyme activity and performed a Student’s *t*-test. Analyses were conducted with R (version 1.2.5033).

## Results

### Global view

**Figure 1.**
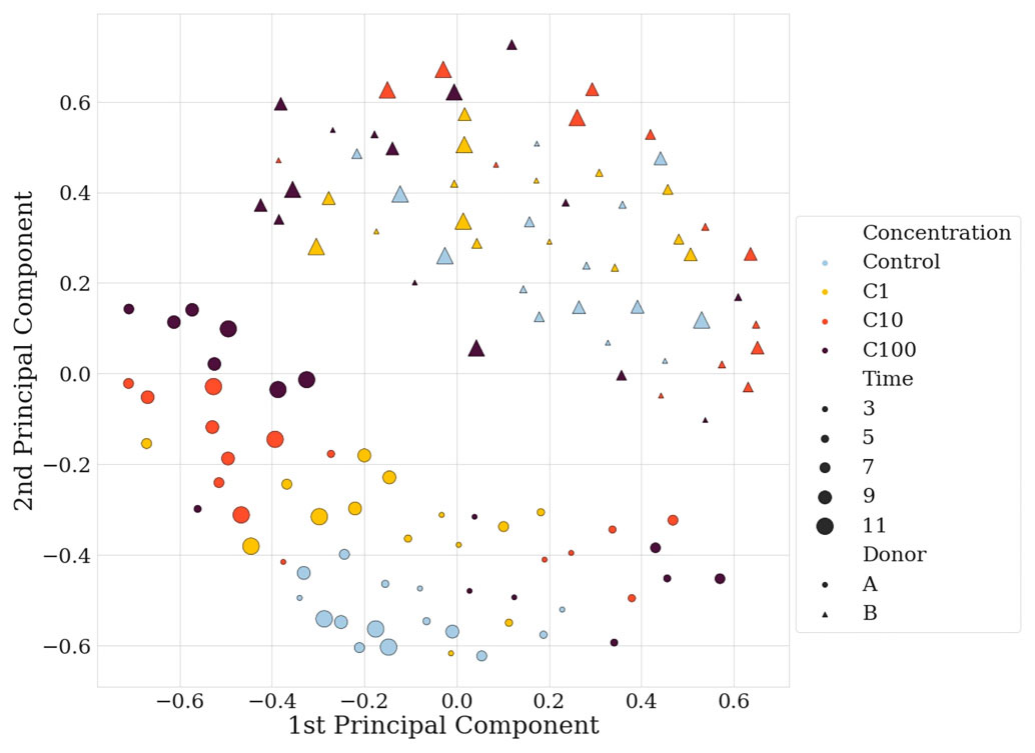
Principal coordinate analysis of the Jaccard matrix, with all donors, all conditions and all timepoints. Dots represent Donor A and triangles Donor B. Size dots/triangles are proportional to the time of the experiment, the bigger the later in the experiment.

Equilibrium state was reached at Day 3, where, for each donor, differences between the chambers (in taxonomy and diversity) were negligible, and all 12 chambers/donor could be considered comparable. There was a minor difference in the baseline Shannon α-diversity between the two donors (ANOVA or PERMANOVA *P *< 0.001) with Donor A having a slightly higher diversity than Donor B (A: 6.65, B: 6.40). Throughout the experiment, the MBRA system preserved each donor’s specificity, which remained easily distinguishable (Figure 1). Indeed, principal coordinate analysis of the Jaccard matrix from all timepoints, all conditions and all donors showed the two donors could well be distinguished throughout the experiment (Figure 1). Colour coding based on concentration revealed the controls of Donor A remained grouped while treatment was associated with a shift, dependent on the concentration (Figure 1). In Donor B, points were spread along PC1 and PC2, with no evident correlation in the time or the concentration of the treatment. This illustrates a heterogenous impact of ceftriaxone, depending on the faecal sample. Although all chambers evolved independently, consistent results were obtained for the two donors. Indeed, all C100 chambers in Donor A shifted in the same manner during the experiment.

### Number of operational taxonomic units (OTUs)

As previously reported, number of OTUs decreased by 2-fold from T0 to T3 corresponding to the stabilization time.^47,51^ After this first period, the number of OTUs remained stable through time for all control chambers (data not shown).

### Impact of the treatment on faecal bacterial communities

Ceftriaxone treatment was associated with a decrease in the α-diversity metrics and an increase in β-diversity distances from baseline, in chambers derived from both donors, but especially in Donor A chambers, in a concentration-dependent manner.

### α-Diversity

**Figure 2.**
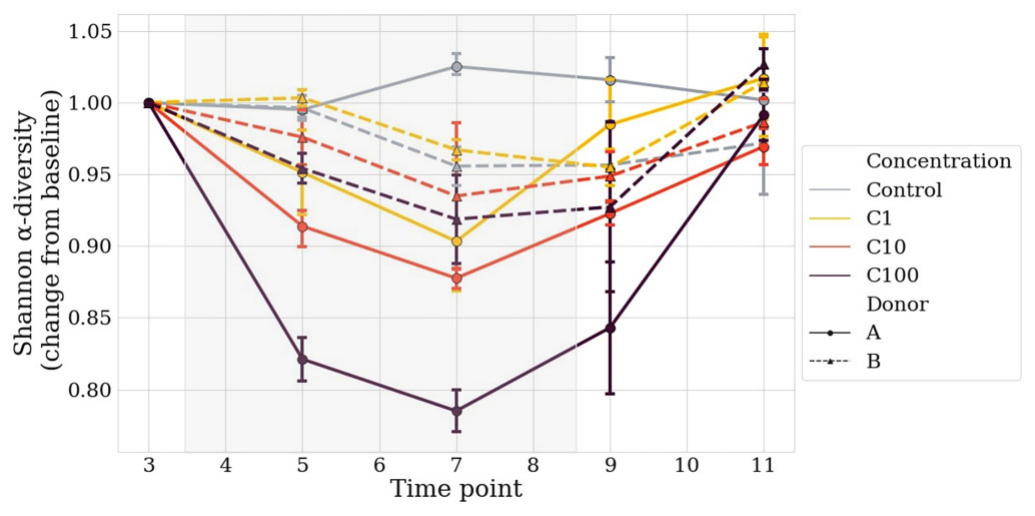
Change from baseline of the Shannon diversity index in Donors A and B, in all conditions. T3 is considered the baseline. Solid lines represent Donor A and dotted lines Donor B. Light blue represents the controls, yellow C1, orange C10 and dark red C100. The grey square represents the treatment period. Average and 68% CI are shown.

The treatment-related disturbance in α-diversity (Shannon index and evenness) started for the majority 24 h after the first dose and reached its maximum after 72 h of treatment (T7). In the control groups, the α-diversity (Shannon index) remained stable in A (T3 to T11) and decreased in B between T5 and T7 (Figure 2). A higher reduction in the Shannon index was observed in Donor A chambers (0.005, 0.097, 0.122 and 0.215 for control, C1, C10 and C100, respectively), compared with Donor B chambers (0.044, 0.045, 0.065 and 0.081 for control, C1, C10 and C100, respectively). This dose-dependent signal was particularly noticeable for Donor A, while it was milder for Donor B. For instance, the lower concentration C1 was almost identical to the controls for the two donors. Interestingly, for both donors, Shannon indexes were comparable to that of the controls 72 h after the last treatment injection, suggesting a fast resilience of the microbiota after the treatment course *in vitro* in the MBRA.

Considering the change from baseline of the distances to T3 for each condition in α-diversity (Shannon index), differences were significant for Donor A (*P* = 8.25 × 10^−5^, two-way ANOVA test), and not for Donor B. Of note, the initial difference of α-diversity observed between the chambers derived from each donor does not seem to explain the difference in the intensities of perturbation associated with treatment. For the highest concentration (C100), at the time of maximum antibiotic impact (T5–T7), the absolute diversity present in microcosm derived from the two donors is inversed: Donor A chambers have lower diversity than Donor B-derived chambers (A: 5.32, B: 5.85, ANOVA or PERMANOVA, *P* = 0.02).

### β-Diversity

**Figure 3.**
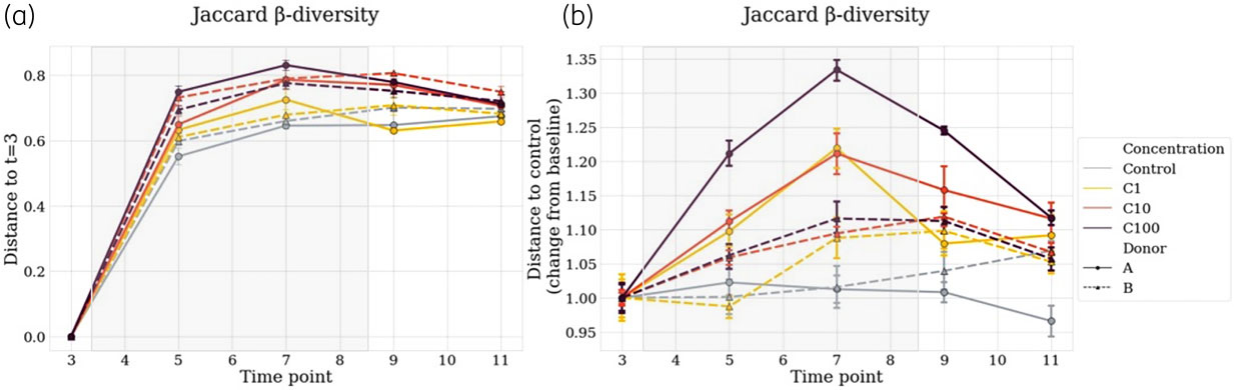
Evolution in β-diversity, in Jaccard metrics. In panel (a), each condition is compared with the control at the same timepoint, and T3 is the baseline. In panel (b), each condition is its own control, including the control group, distance to T3 for each condition is represented. Solid lines represent Donor A and dotted lines Donor B. Light blue represents the controls, yellow C1, orange C10 and dark red C100. The grey square represents the treatment period. Average and 68% CI are shown.

To evaluate β-diversity, we calculated the distance to T3 (stabilization period before treatment) for each condition and the distance to the control chamber between the treated and the control chambers (Figure 3). In this first design, each chamber is its own control, and all timepoints are compared with the T3. In the second design, each chamber is compared with the control chambers (same donor, with the ‘no treatment’ C0 condition) at the same timepoints. Altogether, the first design enabled us to focus on the evolution within each chamber, and the second design enabled us to control for the spontaneous evolution of cultures without treatment in the analysis.

**Figure 4.**
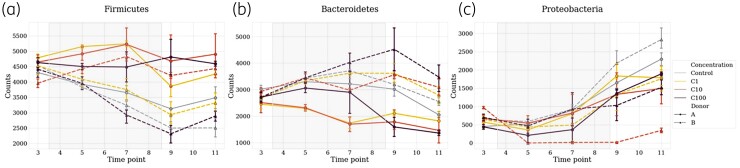
Impact of ceftriaxone on the main three phyla of interest: Firmicutes (a), Bacteroidetes (b) and Proteobacteria (c). Counts were estimated from rarefied tables. Faecal bacterial composition was analysed after 16S rRNA gene sequencing. Solid lines represent Donor A and dotted lines Donor B. Light blue represents the controls, yellow C1, orange C10 and dark red C100. The grey square represents the treatment period. Average and 68% CI are shown.

The bigger the distance, the bigger the change in β-diversity, i.e. the more distinct is the microbiota composition compared with before treatment. At T3, in each donor, all conditions were comparable with a similar distance to the controls. Across time, distance between control and treated groups was correlated with the concentration, for all conditions in Donor A, and particularly for C10 and C100 for Donor B. In both donors, C1 was responsible for a little increase in β-diversity distance compared with the control group. In the treated groups, maximum effect was observed at T7 (Donors A and B), 72 h after the treatment started. With all β-diversity metrics performed here, the effect observed increase in a concentration-dependent manner, C1 being the less impacting concentration, compared with the controls. Considering the change from baseline of the distances to T3 for each condition in β-diversity (Jaccard index), differences were significant in the two donors: A and B, *P* = 0.0128 and *P* = 0.007, respectively (two-way ANOVA test). Decreased distances to the controls were observed after treatment (T11) in both donors, illustrating the gut microbiota resilience after treatment (Figure 4).

### Taxonomic changes

At baseline (T3), in the controls, Firmicutes were dominant (54% of rarefied counts in Donor A chambers and 57% rarefied counts in Donor B chambers). Bacteroidetes (38% rarefied counts in Donor A chambers and 33% rarefied counts in Donor B chambers) and Proteobacteria were less abundant (6% rarefied counts in Donor A chambers and 8% rarefied counts in Donor B chambers) (Figure 4).

**Figure 5.**
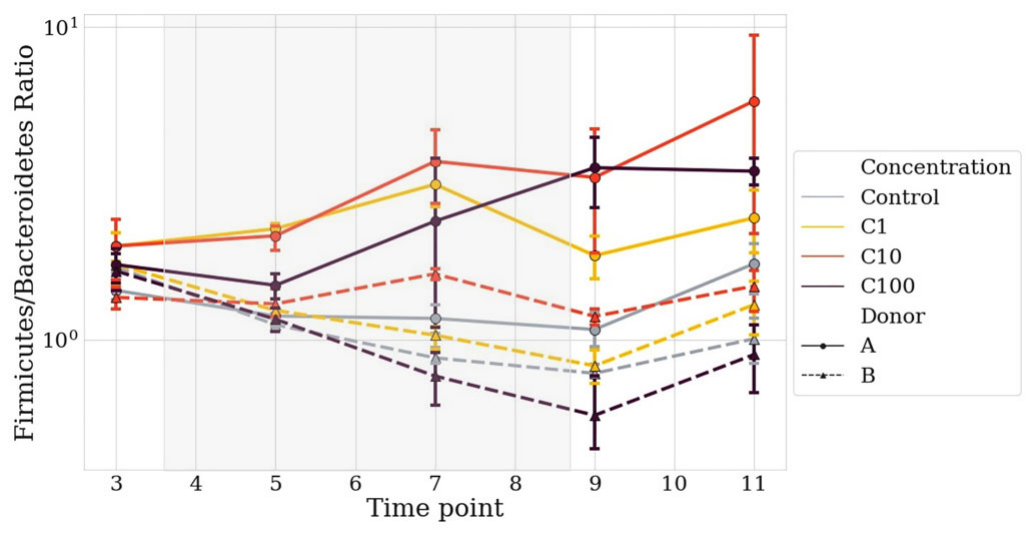
Firmicutes to Bacteroidetes ratio to detect dysbiosis early after treatment. Solid lines represent Donor A and dotted lines Donor B. Light blue represents the controls, yellow C1, orange C10 and dark red C100. The grey square represents the treatment period. Average and 68% CI are shown.

We compared the taxonomic variations at the phylum level, between T3 (stabilization) and T9 (after 5 days of treatment), in the control group and C100 group (Table 1). In Donor A chambers, between the control and the treated group, Firmicutes decreased and increased, respectively; Bacteroidetes remained stable in the control group, and significantly decreased in C100 after treatment. In Donor B chambers, Firmicutes decreased while Bacteroidetes increased in the two groups. To note, Firmicutes and Bacteroidetes behaved inversely between the two donors during the treatment, while Proteobacteria increased in both groups whatever the donor. Drug-associated dysbiosis is more evident in Donor A chambers for which the F/B ratio increases with the treatment (Fisher’s test *P* = 0.0361 in Donor A chambers compared with *P* = 0.897 in Donor B) (Table 1 and Figure 5).

**Table 1.** Variation in Proteobacteria, Firmicutes and Bacteroidetes rarefied counts, between T3 and T9, in Donors A and B, in the control and C100 groups

	Proteobacteria	Firmicutes	Bacteroidetes
Donor A			
Control	+ 263% (*P* < 2.2 ×10^−16^)	− 27% (*P* < 2.2 ×10^−16^)	− 0.03% (*P* = 1)
C100	+ 206% (*P* < 2.2 ×10^−16^)	+ 4% (*P* = 0.003)	− 41% (*P* < 2.2 ×10^−16^)
Donor B			
Control	+ 350% (*P* < 2.2 ×10^−16^)	− 81% (*P* < 2.2 ×10^−16^)	+ 19% (*P* < 2.2 ×10^−16^)
C100	+ 47% (*P* < 2.2 ×10^−16^)	− 84% (*P* < 2.2 ×10^−16^)	+ 67% (*P* < 2.2 ×10^−16^)

Variation in percentage of the mean rarefied counts at T9 and T3, estimate of the *P* value with Fisher’s test.

**Figure 6.**
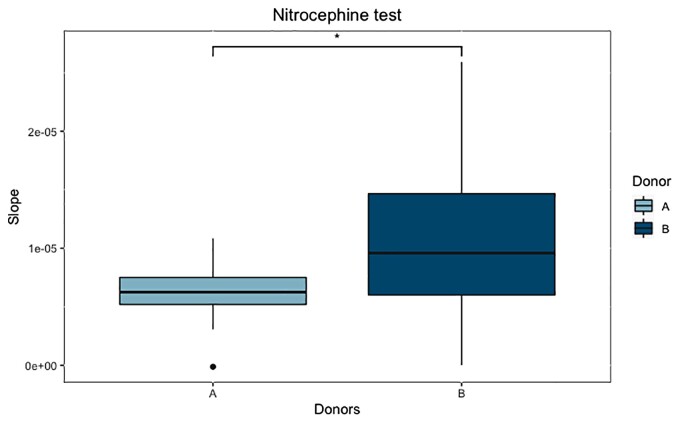
Boxplot representation of β-lactamase activity in Donor A and Donor B, at stabilization time, before treatment, using the nitrocefin test. The *y*-axis represents the slope. The *x*-axis represents the donors. Statistically significant results are represented by an asterisk (Student’s *t*-test, *P* value = 0.035, *n* = 16).

Baseline bacterial β-lactamase activity in the gut has previously been reported to interfere with β-lactam antibiotics. We therefore tested this hypothesis to explain these donor differences in the impact of the treatment. β-Lactamase activity measured at T3 was significantly different between Donor A and B chambers (Student’s *t*-test, *P* = 0.035). Mean and standard deviation of enzymatic activity (assessed as the slope of initial velocity of enzymatic reaction) were 6.25 × 10^−6^ ± 2.55 × 10^−6^ and 9.59 × 10^−6^ ± 6.610 × 10^−6^ in chambers inoculated with Donor A and B, respectively. Donor B chambers had higher β-lactamase activity than those of Donor A (Figure 6).

## Discussion

In this study, we most importantly confirmed the relevance of the MBRA as a tool to study drug–microbiota relations, but we also importantly suggested a microbiological hypothesis to the host-dependent impact of antibiotics on gut microbiota.

To notice, this model is a colon model, in which we concentrate on faecal flora only. Therefore, we do not intend to study the complexity of the human digestive tract, in terms of function, interactions with immune cells, and impact of the treatment on the epithelial cells. Furthermore, we study faecal samples from the lower part of the digestive tract, and therefore our results cannot be generalized to disturbances in other parts of the intestine.

For each donor, all chambers were comparable at the phylum level at inoculation, and stabilization occurred in the same manner in the two groups. Chambers inoculated with each donor’s sample were highly distinguishable throughout the experiment in terms of diversity or microbial composition. The model maintained the host’s specificities, and each culture evolved independently. Intra-host and inter-host specificities were conserved, which is a major characteristic required to conduct faecal culture experiments.^51^

Herein, we add data reinforcing the relevance of the MBRA in culturing human faecal bacterial communities, with the advantage of culturing many samples simultaneously. This high-throughput characteristic gives a non-neglectable advantage compared with *in vivo* studies.

To test the impact of an antibiotic treatment in the MBRA, we chose to study ceftriaxone, a third-generation cephalosporin with a long half-life.^61^ Like most β-lactams it is a time-dependent killer. It is widely used for its broad spectrum of activity and is administered once daily, explaining our experimental design here. Ceftriaxone is mainly eliminated in the urine^33^ and in the bile (11%–65% of the dose infused).^62,63^ Due to saturable tubular reabsorption, and saturable protein binding (albumin), concentration of free drugs and biliary concentrations of ceftriaxone^62–64^ are heterogenous between individuals (up to 3-fold difference), explaining variations in excretion and thus variable concentrations of the drugs in the gut.^33,61–64^ Indeed, the biliary excretion of ceftriaxone is directly linked to the impact of the molecule on faecal microbiota, adding evidence in the link between biliary clearance parameters and the importance of drug-associated disturbance in the faecal microbiota.^33,64^ Finding data on the concentration of ceftriaxone in the faeces is therefore challenging. Indeed, the concentration of ceftriaxone ranged from indetectable to over 1000 mg/kg of faeces in healthy humans, after 5 days of treatment with 1 g daily.^65^ To circumvent these concentration variations between hosts, we chose to study three different concentrations of ceftriaxone that are based on peak concentrations from *in vivo* data (we tested 10 times more and 10 times less). The maximum tested concentration in our study, corresponded to the plasmatic peak in healthy individuals after 1 g of ceftriaxone.^64,66^ Similar to the literature, and to mimic a common treatment course, we evaluated a 5 day course of treatment^65^ and found a slow return to baseline from 72 h after the last dose.

After treatment with ceftriaxone, in healthy patients, *in vivo* studies report a decrease in Enterobacteriaceae, *Escherichia coli*, staphylococci, streptococci, lactobacilli and *Bifidobacterium*, an increase in enterococci^5,22,62,65^ and an overgrowth of yeast.^33,65,67^ On a larger scale, a reduction in Bacteroidetes along with an increase in Firmicutes has been described after broad-spectrum antibiotic treatments,^13^ such as third-generation cephalosporins. When comparing our results with a similar study performed *in vivo*,^66^ we also observed an important disturbance in the overall diversity of the faecal microbiota (decrease in the Shannon index, an increase in β-diversity metrics with the same kinetics), associated with changes in the composition of the faeces. These results confirm the reliability of our experiment, in which we observed the same tendencies in taxonomic changes.

As an early and easy screening tool for dysbiosis, the F/B ratio has been mentioned in literature.^68–70^ An increase in the F/B ratio seems to be associated with an unhealthy state.^68^ Our experiment allowed us to measure this ratio, confirming its disturbance was related to the treatment period, and shift occurs first among the other monitored parameters (diversity and taxonomy). Mild variation was observed in the control groups but neglectable compared with the important fluctuation in the treated groups. This ratio is easy to measure and should be further evaluated as an early dysbiosis diagnostic tool.

However, it is important to mention that, depending on the disturbed species, the F/B ratio can remain stable while dysbiosis occurs in the gut microbiota. It is therefore a simple marker to measure but has limitations that should be well known. This is of major importance since some species are responsible for the loss of colonization resistance of the gut microbiota.

Colonization resistance has not been clearly studied here but it is one of the further issues we will work on. Indeed, in an *in vitro* gut model, Rooney *et al*.^39^ have already reported a clonal expansion of carbapenemase-producing strains after antibiotic treatment, and described resistance gene transfer between species from faecal samples from healthy donors. Further studies must be conducted to better understand the underlying risks of such antibiotic-associated dysbiosis in the colonization resistance of the gut microbiota.

The dose dependent signal we observed in our experiment well illustrates the sensitivity of the MBRA to this pharmacokinetic parameter, widening the experimental options in the future. In addition, this model preserves host specificity, offering the advantage of personalized studies. Indeed, Donor A seemed more permeable to treatment-induced modifications, compared with the Donor B, in terms of α-diversity. The marked difference in treatment effects between the two donors suggests that microbiota can influence the consequences of antibiotic treatment, and that may be an important factor for the spread of antibiotic resistance. Here, though limited to only two donors balanced by a robust number of replicates, our study suggests that the microbiota may contribute to this variability of response.

Resilience of the faecal flora in our study was observed earlier after the disruption of treatment. *In vivo* resilience is often observed after several days to weeks after the end of the treatment.^26^ This difference could be explained by the simplicity of the *in vitro* experiment compared with the complexity of human functions. In our system, bacteria can grow if the culture conditions are optimal for bacterial growth, provided there are no other factors that interfere with the bacterial growth. In humans, bacterial growth also depends on other exogenous stress conditions that bacteria are exposed to.

Similarly to previous findings,^71^ we quantified the β-lactamase activity of the microbiota. A stronger β-lactamase activity in Donor B chambers could directly explain the reduced impact of the ceftriaxone on the faecal microbiota, compared with Donor A chambers. Indeed, numerous anaerobic bacteria produce cefuroximase or cephalosporinase enzymes whose spectrum affects ceftriaxone. Among these bacteria, many belong to the Bacteroidetes phylum, increasing in the Donor B chambers during treatment, while decreasing during the same period in the Donor A ones. A stronger β-lactamase activity suggests the capacity of this microbiota to hydrolyse β-lactams and thus protect the bacterial community from the impact of ceftriaxone.^71^ The heterogenous impact of this molecule on the gut microbiota well illustrates the ‘host-specific’ response to a changing environment, reinforcing the importance of *ex vivo* models to conduct more personalized studies.^71^ To go further, this host-dependent effect is a major parameter to consider when studying the dynamics of antibiotic resistance.^31^ Gut microbiota resistance to treatment-induced dysbiosis is well illustrated here for Donor B. Furthermore, we suppose here the adaptability of the gut microbiota to environmental pressure with an increase in the bacterial phylum implicated in the production of the enzyme hydrolysing the treatment, therefore increasing the protection against dysbiosis.

### Conclusions

Ceftriaxone impacts the richness of the microbiota, the α- and β-diversity, and the reported taxonomic changes observed are consistent with those described *in vivo*. Disparities between donors could be explained by a different microbial composition and different β-lactamase activity that could participate, by its altruistic effect, to protect the bacterial community. The MBRA model enabled us to reliably mimic *in vitro* the impact of ceftriaxone on human faecal bacterial communities, in a rapid and reliable manner. The capacity of this system to preserve the specificities of each culture chamber and to distinguish easily different donors is of major interest to study host-dependent variations in antibiotic resistance dynamics, or more largely to study the interaction between drugs and gut microbiota, in a personalized approach.

## Data Availability

The datasets during and/or analysed during the current study available from the corresponding author on reasonable request.
